# Evaluation of interaction between *Piriformospora indica*, animal manure and NPK fertilizer on quantitative and qualitative yield and absorption of elements in sunflower

**DOI:** 10.1002/fsn3.1571

**Published:** 2020-05-12

**Authors:** Siamak Eliaspour, Raouf Seyed Sharifi, Ali Shirkhani

**Affiliations:** ^1^ Department of Agronomy and Plant Breeding Faculty of Agriculture University of Mohaghegh Ardabili Ardebil Iran; ^2^ Crops and Horticulture Research Department Kermanshah Agricultural Resources Research and Education Center (AREEO) Kermanshah Iran

**Keywords:** chemical fertilizer, manure, *Piriformospora indica*, sunflower

## Abstract

Microbial endophytes are considered as one of the most important soil microorganisms which increase their yield per unit area by generating genetic, physiological, and ecological changes in their host plants. We conducted an experiment as factorial in a completely randomized manner with three replications at the Ghasre Shirin greenhouse of Kermanshah province in order to evaluate the interaction of *Piriformospora indica* (inoculation–noninoculation) with manure (25% of the flowerpot volume—without using manure as a control) and NPK chemical fertilizer (10 g per flowerpot—without the use of fertilizer as a control), the quantitative and qualitative yield and absorption of elements in sunflower. The results showed that the interaction of *P. indica*, manure, and chemical fertilizer on the colonization percentage, seed oil percentage, nitrogen concentration, phosphorus concentration, and the 1000‐seed weight was significant at 1% probability level, and on yield index, growth, plant height, and concentration of potassium element it was at 5% probability level. Bilateral effects of *P. indica* with chemical fertilizer and the manure treated with chemical fertilizer on the budding time were significant at the probability level of 1% and 5%, respectively. It seems that the coexistence between sunflower root and *P. indica* increases the growth of the root system of the plant; thereby it increases the height of plant because of the absorption of essential elements such as nitrogen, phosphorus, and potassium by the root, and increases the quantitative and qualitative yield of sunflower.

## Graphical abstract

Evaluation of interaction between biological fertilizer (*Piriformospora indica*) manure and NPK fertilizer on the qualitative and quantitative yield and elemental uptake of sunflower

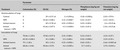

## 1 INTRODUCTION

Due to some environmental and economic constraints, the use of chemical fertilizers is not a good solution for the food shortage, especially in developing countries such as Iran (Gallego, Benavides, & Tomaro, 1996; Jahanbakhshi & Kheiralipour, 2019). Nowadays, the strategy for nutrition improvement has been changed. Reduction of the use of chemical fertilizers is considered as an important principle in agriculture (Varma et al., 2015). Increasing the absorption and availability of nutrients of plants using the microorganisms is considered as one of the important goals of soil fertility management in pursuit of sustainable agriculture. Endophytic fungi, are as one of the most beneficial microorganisms in the soil, by making ecophysiological changes in host plants, while increasing their yield per unit area, allow the development of plant cultivation in soils with adverse environmental and nutritional conditions (Babaei Ghaghelestany, Jahanbakhshi, & Taghinezhad, 2020; Lindahl et al., 2007). Viscous‐arbuscular mycorrhizal fungi are considered as beneficial fungi that improve plant growth by providing a wider absorbent surface for the transfer of nutrients in the root to plants. The benefits of this linkage include the production of a variety of hormones which stimulate plant growth (including auxin and cytokinin), increasing product yield, increasing plant resistance to pathogenic agents, help reducing environmental stresses (heat, salinity, soil contamination to toxins or Heavy metals) and, most importantly, reducing the use of chemical fertilizers (Krich et al., 2000). The Piriformospora indica (*P*. *indica*) fungus has a wide range of host plants and stimulates the growth of its host by colonization of the root. *P. indica* establishes a colonization relationship with a large number of flowering plants (Monocotyledon and Dicotyledon) (Singh, Sharma, Rexer, & Varma, 2000). The effect of inoculation of *P. indica* on increasing the life of plants such as corn, tobacco, parsley, artemisia, and poplar has been reported (Verma et al., 1998). The effect of this fungus on chickpea, peas, beans, and soybeans has also been proven (Rai, Acharya, Singh, & Varma, 2001; Rai & Varma, 2005). Inoculation of Chlorophytum borivilianumL. seedlings with *P. indica* fungi has improved the seedling survival, chlorophyll, phosphorus, and iron concentration (Gosal, Karlupia, Gosal, Chhibba, & Varma, 2010) and has increased zinc absorption in lettuce (Padash, Shahabivand, Behtash, & Aghaee, 2016). The effect of *P. indica* is positive on the adsorption of elements such as phosphorus in plants (Yadav et al., 2010). Another use of chemical fertilizer is in manure. The presence of organic matter improves the physical, chemical, and biological conditions of the soil. Minerals can be dissolved in water and can be exchanged in the soil, or they can form part of the organic matter to be slowly released and used in the plant. As a result, erosion and loss of soil will be minimized (Manna, Swarup, Wanjari, Mishra, & Shahi, 2007). In investigating the effects of organic and chemical fertilizers on yield, yield components and qualitative characteristics of sunflower, Shyalaja and Swarajyalakshmi (2004) showed that the grain yield was significantly influenced by the combined treatment of manure, poultry manure along with the nitrogen concentration chemical fertilizer. The reason is more access to the food needed during the critical stages of plant growth. The combined system of organic and chemical fertilizer has been reported in other researches on sunflower (Munir, Malik, & Saleem, 2007) and peanut (Basu, Bhadoria, & Mahapatra, 2008). Shehata and El‐Khawas (2003), in the study about the effect of biological manures on growth parameters and the yield of sunflowers, found that fertilizers improve quality and yield compared to inoculum. Due to mass production of sunflower in the country and excessive use of chemical fertilizers, the present study was conducted to prevent overuse of chemical fertilizers and to replace *P. indica* with chemical fertilizers and also increasing yield, growth, increasing oil content, and nutrient uptake in sunflower plant was treated with P. indica endophyte and organic fertilizer and chemical fertilizer.

## 2 MATERIALS AND METHODS

The present study was conducted to evaluate the effect of *P. indica* (inoculation–noninoculation), manure (25% of the 50‐kilos flowerpot, nonuse), and chemical fertilizer (10 g as an equal amount of nitrogen, phosphorus, and potassium, nonuse) on the yield, yield components, and percentage of sunflower oil of Varroflor cultivar were studied as factorial in a completely randomized manner with eight treatment and three replications in a greenhouse in Ghasre Shirin of Kermanshah province in 2017. The soil was first placed in the oven in a temperature of 70°C for 24 hr to eliminate all existing microorganisms to begin the experiment. The results of soil and manure tests are presented in Tables 1 and 2.

**TABLE 1:** Shows physical and chemical properties of tested soil

K (mg/kg)	P (mg/kg)	*N* (%)	O.C (%)	EC (dS/m)	Acidity (pH)
597	15	15	1.36	0.63	7.05

**TABLE 2:** Specimens of manure

PH	Organic carbon	Total nitrogen	Total phosphorus	Total potassium
9	28.85	2.55	0.56	1.25

### 2.1 Treatments

1. Noninoculation of *P. indica* + nonuse of manure + no use of fertilizer
2. Noninoculation of *P. indica* + nonuse of manure + chemical fertilizer (10 g per flowerpot)
3. Noninoculation of *P. indica* + fertilizer (25% of per flowerpot) + nonuse of fertilizer
4. Noninoculation of *P. indica* + manure (25% of flowerpot) + fertilizer (10 g per flowerpot)
5. Noninoculation of *P. indica* + nonuse of manure + nonuse of fertilizer
6. Inoculation of *P. indica* + nonuse of manure + chemical fertilizer (10 g per flowerpot)
7. Inoculation of *P. indica* + manure (25% of flowerpot volume) + nonuse of chemical fertilizer
8. Inoculation of *P. indica* + manure (25% of flowerpot) + chemical fertilizer (10 g per flowerpot)

Five bushes were planted in a 50 kg plastic flowerpot with top diameter of 35 cm. The number of plants was reduced to 3 plants per flowerpot after planting. Uroflor oil sunflower cultivar is the cultivar which has been used. The minimum and maximum temperatures were 28°C and 14°C, respectively, and relative humidity was about 55% up to 60%. Sunflower seeds were also exposed to 14 hr of light (a combination of Fluorescent and Tungsten lumens). Strain of *P. indica* was obtained from the Department of Horticulture of Maragheh University and was used as seed priming. After 1 month (shahabivand, Parvaneh, & Aliloo, 2017), samples of thin roots of sunflower were isolated and the colonization percentage was measured using the Phillips and Hayman (1970) method and the following equation (Equation 1).

(1) $$\begin{array}{cc} & \text{Measuring the coexistence percentage}\;\\ & \text{Percentage of colonization}=\frac{\text{Number of points with mycorrhizal colonization}}{\text{Total number of points}}\times 100 \end{array}$$

The final harvest (88 days after planting), the final height of the plants, the diameter of the head, and the grain yield were measured. Percentage of seed oil was also measured using the Soxhlet extractor and Diethyl ether solution (Eyvazzadeh, Seyyedain Ardebili, Chamani, & Darvish, 2010). Values of elements and phosphorus by spectrophotometry on 420 nm wave length, potassium using dry ashing method and flame photometer, and nitrogen were measured by Kjeldahl method. The phonological stages, such as the emergence of the flower head based on the number of days from the planting to the emergence of the flower head per plant in each flowerpot, flowering stage based on the number of days from the planting to viewing the yellow flowers and the physiological maturation of seeds, were determined by seeing the change of color from green to yellow. Each step was determined as the day growth rate in GDD, using the following equation (Equation 2).

(2) $$\text{GDD}=\frac{\text{minimum temperature}+\text{maximum temperature}}{2-\text{base temperature}}$$

Data were analyzed using SAS 9.1 software. A comparison of the mean of data was done using Duncan's multi‐domain test at the 5% probability level. Graphics were designed using software (Abbaspour‐Gilandeh, Kaveh, & Jahanbakhshi, 2019; Jahanbakhshi, Ghamari, & Heidarbeigi, 2017; Jahanbakhshi & Salehi, 2019).

## 3 RESULTS AND DISCUSSION

The results of the variance of data analysis (Table 3) showed that the effect of *P. indica*, animal manure, and chemical fertilizer on root colonization, phosphorus concentration, oil percentage, and nitrogen percentage of the grain was meaningful at a probability level of 1% and on potassium concentration at 5% probability level.

**TABLE 3:** Analysis of variance of the influence of *P. indica*, animal manure, and chemical fertilizer on concentration of nitrogen, phosphorus, potassium, and oil of sunflower

Sources of changes	Average of squares					
	Df	Colonization percentage	Nitrogen	Phosphorus	Potassium	Oil
Fungi	1	28,912.04**	0.131**	0.234**	0.006 ns	176.04**
Animal manure	1	70.04**	0.009**	0.243**	0.048 ns	63.37**
NPK	1	3.37 ns	0.778**	22.62**	1.68*	198.37**
Fungi × NPK	1	3.35 ns	0.001 ns	0.14**	0.001 ns	7.04**
Fungi × animal manure	1	70.04**	0.001 ns	0.031**	0.111 ns	5.04 ns
Animal manure × NPK	1	51.04**	0.025**	0.003**	0.005 ns	0.37 ns
NPK × animal manure × fungi	1	51.002**	0.013**	0.002**	0.018*	22.04**
Error	14	5.87	0.001	0.125	0.008	2.042
Coefficient of variation (%)	—	14.2	12.2	5.5	21	18.3

Abbreviation: ns, nonsignificant.

* Significant at a probability level of 5%.

** Significant at a probability level of 1%.

### 3.1 Percentage of colonization

According to the table of the comparison of averages (Table 4), the highest percentage of colonization was observed for the plants which were treated with P. indica and animal manure, and the lowest amount of colonization was related to inoculated plants or fungi with chemical fertilizer. Studies on tomatoes have shown that the percentage of colonization is reduced by increasing levels of chemical fertilizers in the plant. Pawlowski and Charvat (2004) showed that Arbuscular mycorrhizal (AM) spore is highly sensitive to being placed in an environment in which the concentration of chemical elements is higher. They concluded that the susceptibility of fungal spores to these metals (nitrogen, zinc, manganese, and cadmium) may be due to the saturation of the cellular system responsible for buffering metals, while placed in saturation conditions which were similar to the results of this study. Reducing the degree of colonization in the sunflower root with increasing the concentration of the elements may be due to increasing the growth of the poisonous properties of elements in high concentration, which reduces the potential of inoculum, and prevent the budding and natural growth of the spore. Despite of reducing the colonization in high concentrations of the elements (Table 4), simultaneous use of fungi and chemicals is very useful to increase the yield and other growth parameters.

**TABLE 4:** Comparison of the effect of *P. indica*, animal manure, and chemical fertilizer on percentage of colonization and the amount of nitrogen, phosphorus, and potassium in sunflower plant

Treatment	Parameter				
	Colonization (%)	Oil (%)	Nitrogen (%)	Phosphorus (mg/kg net weight)	Potassium (mg/kg net weight)
Noninoculation of fungi					
Control	0	39 ± 0.57 cd	2 ± 0.004 g	0.18 ± 0.008 g	2.03 ± 37/0 c
NPK	0	33.66 ± 0.88 e	2.11 ± 0.008 f	0.31 ± 0.23 f	2.12 ± 32/0 c
Animal manure	0	40 ± 0.78 cd	4.01 ± 0.004 d	0.45 ± 0.08 d	2.6 ± 01/0 ab
Animal manure × NPK	0	43.66 ± 0.87 b	4.5 ± 0.008 b	0.65 ± 0.01 b	2.56 ± 41/0 ab
Inoculation of fungi					
—	70.66 ± 1.20 b	37.66 ± 0.57 d	2.31 ± 0.88 e	0.12 ± 0.01 h	2.14 ± 0.27 c
NPK	62.33 ± 0.88 d	41 ± 0.88 c	2.3 ± 0.88 e	0.32 ± 0.008 e	2.14 ± 0.55 c
Animal manure	75 ± 1.20 a	50 ± 1.15 a	4.31 ± 0.006 c	0.62 ± 0.01 c	2.66 ± 0.005 ab
Animal manure × NPK	68.66 ± 3.46c	40.66 ± 0.57c	4.6 ± 0.005 a	0.7 ± 0.01 a	2.74 ± 0.043 a

### 3.2 Concentration of NPK elements

Nitrogen, potassium, and phosphorus concentration in seeds showed that the highest percentage of nitrogen, phosphorus, and potassium concentration was related to fungal treatments with manure and fertilizer in inoculated plants with *P. indica* (Table 4). It seems that the absorption of elements is high around the root due to the effect of colonized fungus on increasing root growth (Padash et al., 2016). Alizadeh (2010) stated that the absorption of nitrogen, phosphorus, potassium, and magnesium in the plant has been increased and iron absorption has been decreased by increasing fertilizer application. Staal, Maathuis, Elzenga, Overbeek, and Prins (1991) stated that increasing the absorption of cations is one of the positive effects of using NPK. Therefore, nitrogen absorption by the plant also increases the relative absorption of other nutrients. Animal manure creates an appropriate ground for the growth of the root and increases the vegetation growth of plants by providing many nutrients, building up the soil structure, and also increasing the capacity to maintain moisture (Ahmadian, Ghanbari, & Galavi, 2004). According to various scholars, the use of animal manure increases the organic substance, useable phosphorus (Antoun, Beauchamp, Goussard, Chabot, & Lalande, 1998), nitrate nitrogen (Graham & Vance, 2000), and other nutrients of the plant, and improves soil texture (Sharpley, McDowell, & Kleinman, 2004), which ultimately increases the quality of product slightly. According to the results of the test and the results of previous reports, it seems that manure mixed with soil plays a significant role in the growth of fungus on roots. It also provides useful elements for the plant.

### 3.3 Oil percentage

Comparison of averages showed that application of manure in inoculated plants with P. indica increased the percentage of seed oil (25%) (Table 4) and the lowest amount of oil was related to the application of chemical fertilizers (Table 4). The results of this study also showed that application of manure fertilizer with mycorrhizal fungi increased seed oil percentage in sesame, in such a way that the simultaneous application of compost, vermicompost, and sulfur granules increased the amount of sesame oil by 13%, 12%, and 10%, respectively, compared to the separate application of mycorrhiza (Rezvani Moghaddam, Sabori, & Mohamadabadi, 2015). Munir et al. (2007) investigated various fertilizer systems on sunflower plant, which showed that the combined treatment had the lowest percentage of oil, and the control treatment had the highest oil concentration. The reduction of oil concentration with the high application of nitrogen fertilizers has been reported by other researchers (Khaliq, 2004). Kasem and EL‐Mesilby (1992) reported that the percentage of seed oil decreases by increasing access to nitrogen. (Steer & Seiler, 1990) also found that there is a negative relationship between the amount of access to nitrogen and the amount of oil. Application of biologic fertilizers with organic fertilizers increased the amount of oil. Therefore, the *P. indica* fungi which has been combined with manure increases the amount of seed oil in the sunflower.

According to analyzing the table of variance (Table 5), it was determined that application of *P. indica* fungus with manure and chemical fertilizer on 1000‐seed weight was significant at the probability level of 1% and for plant height, growth time, and yield at 5% probability level. In addition, the treatment effects of *P. indica* with chemical fertilizer and animal manure on the appearance of sunflower head were significant at 1% probability level. Treatment of *P*. *indica* and animal manure was significant at 5% probability level on head diameter indexes. The effect of animal manure and chemical fertilizer on the diameter of head and head emergence was significant at 1% probability level.

**TABLE 5:** Shows the analysis of variance of treatment with *P. indica*, animal manure, and chemical fertilizer on grain yield, heading, growth, height, and head diameter of sunflower

Sources of changes	Average of squares						
	Df	1000‐seed weight	Height	Growth	Head diameter	Heading	Yield
Fungi	1	223.32**	828.37**	228.16**	3.39**	130.66**	5,441.69**
Animal manure	1	475.17**	900.37**	66/66**	1.34*	88.16**	5,071.69**
NPK	1	1697.64**	950.04**	5.253**	7.43**	793.5**	16,142.5**
Fungi × NPK	1	25.03**	92.04 ns	0.66 ns	0.01 ns	66/66**	20.53 ns
Fungi × animal manure	1	131.27**	108.37*	4.16 ns	1.51*	10.66 ns	03/30 ns
Animal maure × NPK	1	52.48**	117.04*	1.5 ns	1.26*	37.5*	1,378.25*
NPK × animal manure × fungi	1	50.66**	117.04*	16.66*	0.42 ns	0.2 ns	1906.14*
Error	14	2	24.08	2.25	0.173	4.667	247.88
Coefficient of variation (%)	—	8.7	13	14.15	17	13.3	11.10

Abbreviation: ns, nonsignificant.

* Significant at a probability level of 5%.

** Significant at a probability level of 1%.

### 3.4 Growth

Comparison of the meanings the effect of symbiotic fungus treatment with manure and chemical fertilizer on head maturity index in sunflower (Table 5) showed that the plants which were treated with colonization fungus, animal manure, and chemical fertilizer completed their growth stage sooner than other plants. It seems that the availability of these plants to nutrients, especially nitrogen, at an early stage of growth is due to their rapid growth. On the other hand, the appropriate texture of the soil because of the presence of manure and the better root yield of the plant through the P. indica fungus cause more elements to be absorbed at the beginning of the growth stage and the plant cultivates quickly its vegetative and reproductive phase. It is likely that the biological fertilizer used in this study and the mechanism of growth stimulating hormones have decreased the vegetative growth period during the experiment. Marius, Octavita, Eugen, and Vlad (2005) stated that sunflower mycorrhizal inoculation improves the photosynthesis process, production of energy and, consequently, growth of sunflower in comparison with the nonuse of biological manure because of the increased activity of catalase and the concentration of chlorophyll a and b. The *P. indica* fungus accelerates seed germination and rapid absorption of the elements by secreting the auxin hormone. It is likely that the absorption of most of the elements, especially nitrogen, in the early stages of growth will lead to the emergence of the vegetative phase. The chemical fertilizer around the root can be easily absorbed by the plant and thus cause rapid growth because of the high efficiency of the roots of inoculated plants with fungi.

### 3.5 Height

Comparison of averages showed that the height of plants colonized with *P. indica* in combination with manure and chemical fertilizer had a significant difference compared to noninoculated plants (Table 6). (Skinner, Boddey, & Fendrik, 1987) reported that inoculation of seed with biological fertilizers increases root development and absorbs water and nutrients better. This can improve plant growth and increase plant height. The P. indica fungus makes most of the elements, especially nitrogen, to be absorbed with the effect on the root system. Animal manure provides a good basis for the presence of elements around the root, makes the plant to grow better, and is effective in plant growth and height. Khoramdel, Kochaki, Nasirimahalati and Ghorbani, (2008) confirmed the effect of biological fertilizers on the height of black caraway plant, and Akbari et al. (2002) confirmed the role of biofertilizers in increasing sunflower plant height.

**TABLE 6:** Comparison of the effects of endophytic fungus *Piriformospora indica*, animal manure, and chemical fertilizer on growth indices and yield in sunflower plant

Treatment	Parameter			
	Height (cm)	Grain yield (kg per hectare)	Growth	1000‐seed weight
Noninoculation of fungi				
Control	108.66 ± 1.45 c	3,317.48 ± 8.41 c	2075.33 ± 0.66 a	34.38 ± 0.48 g
NPK	116.66 ± 2.08 b	3,333.3 ± 11.02 c	2066.33 ± 0.66 bc	36.66 ± 0.48 f
Animal manure	117.03 ± 1.76 b	3,364.83 ± 8.81 ab	2066.02 ± 0.66 bc	49.10 ± 0.73 c
Animal manure × NPK	124.33 ± 2.08 b	3,420 ± 12.83 a	2061.33 ± 0.66 d	49.66 ± 0.84 c
Inoculation of fungi				
—	116.66 ± 2.02 b	3,341.65 ± 12.7 cb	20.69 ± 0.66 b	38.55 ± 0.33 e
NPK	124.33 ± 1.45 b	3,397.59 ± 5.25 a	20.65 ± 0.66 c	44.68 ± 0.88 d
Animal manure	125.33 ± 2.08 b	3,394.34 ± 4.95 a	2064.33 ± 0.66 c	53.38 ± 0.14 b
Animal manure × NPK	149.66 ± 1.45 a	3,418.33 ± 3.28 a	2057.66 ± 0.66 e	69.1 ± 0.48 a

### 3.6 Grain yield

Grain yield in plants which was treated with *P. indica* was not significantly different from the control plants (noninoculation) (Table 6). However, the highest yield belongs to the use of fungi, along with animal manure and chemical fertilizer (Table 6). Probably, improving the physical properties of soil, better access to water and nutrients, also the ease of absorption of elements are the reason for increasing sunflower seed yield in manure (Mallanagouda, 1995). The introduction of organic matter into soil increases soil nutrient uptake and absorption capacity by the plant (Beare, 1997). The results of (Liang et al., 2005) showed that the manure provides a suitable ground for plant growth and ultimately boosts yield because of the presence of elements and water around the root.

### 3.7 1000‐seed weight

Comparison of mean values showed that the highest 1000‐seed weight belonged to P. indica‐inoculated plants with cadmium and chemical composition (Table 6). Studying the levels of nitrogen fertilizer on the yield of sorghum, Powell and Hons (1992) stated that there is a direct relationship between nitrogen fertilizer application and grain yield, the production of biomass and the weight of a 1000‐seed weight. They attributed the increasing weight of 1,000 seeds and dried substance, to the increase of chlorophyll concentration, photosynthesis, and photosynthesis substances. Asare and Scarisbrick (1995) reported that consumption of 240 kg of nitrogen per hectare, combined with animal manure and biological fertilizers, has increased the 1000‐seed weight, grain yield, and dry matter concentration of the rapeseed. Cheema, Malik, Hussain, Shah, and Basra (2001) stated that consuming 135 kg of pure nitrogen per hectare had the greatest effect on grain yield, grain yield components, and percentage of produced oil. The more the plant growth and absorption of elements, especially nitrogen, increases, the more the weight of 1,000 seeds will increase in the plant. In the table of the comparison of averages (Table 4), the highest percentage of nitrogen was associated with the plants colonized with *P. indica,* which was used in combination with chemical fertilizers and animal manure. Considering that fertilizer has significant amount of nitrogen, it is estimated that the plants, which are treated with this fertilizer, should have 1000‐seed weight more than other plants. On the other hand, the colonization of the plant with the combination of manure and chemical fertilizers increases the absorption of these elements.

### 3.8 Head diameter

**FIGURE 1:**
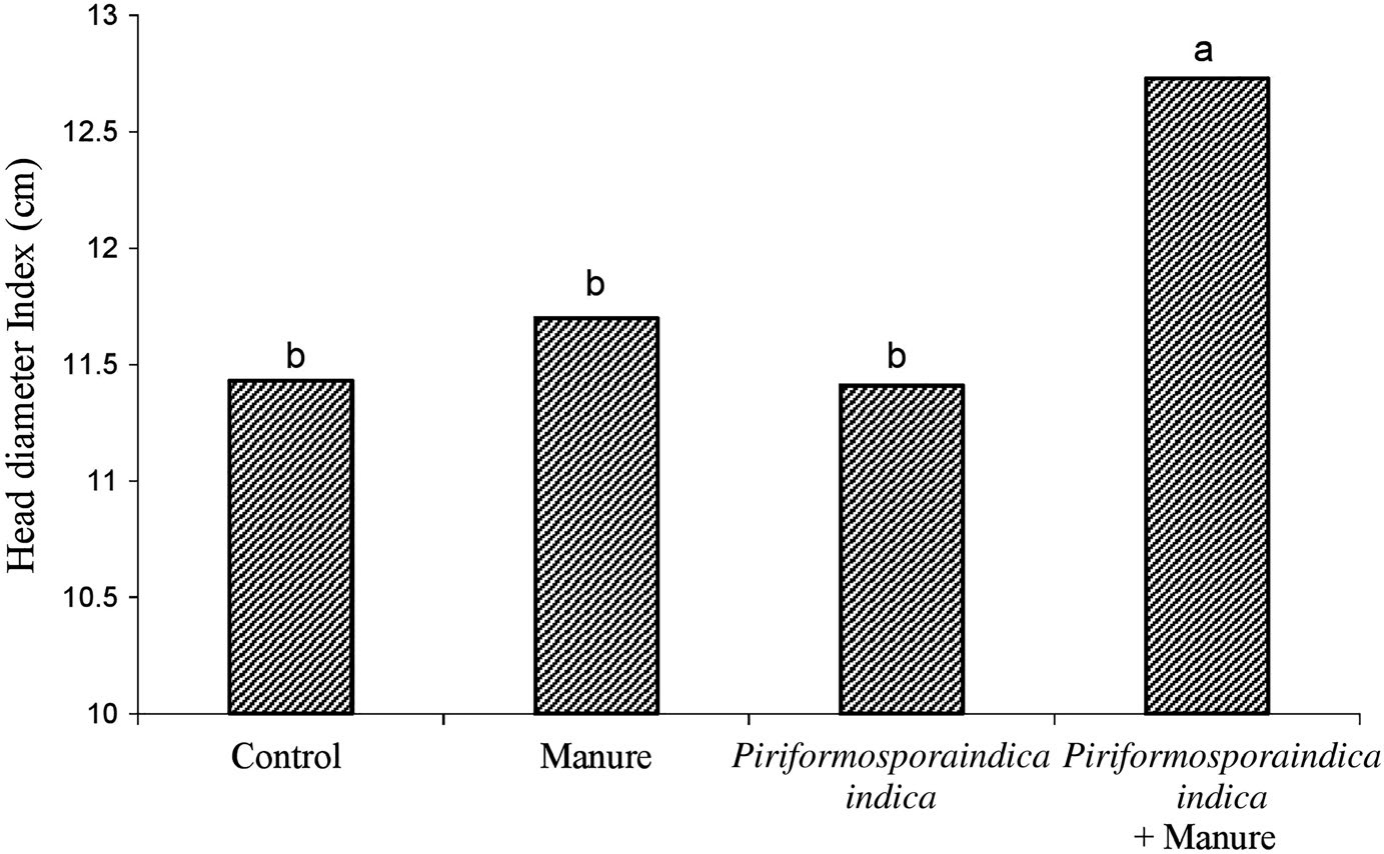
Shows the effect of *Piriformospora indica* and manure on the head diameter index of the sunflower plant

**FIGURE 2:**
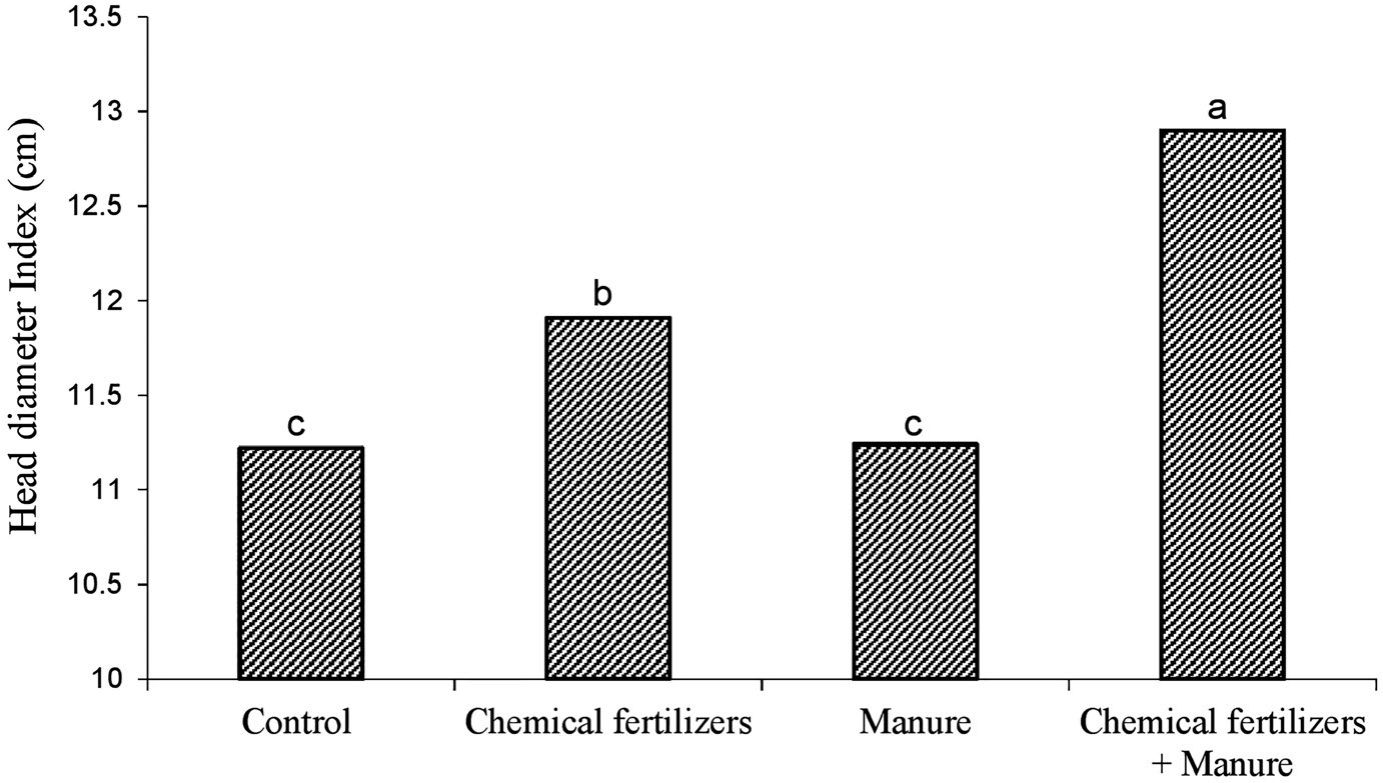
Shows the effect of chemical fertilizer and manure on the diameter of head of sunflower plant

Comparison of averages showed that the combination of P. indica fungi with animal manure (Figure 1) and manure with chemical fertilizer (Figure 2) has the greatest effect on head diameter. The results of various experiments showed that the use of some elements in sunflower production has a significant effect on plant height, head diameter, seed number, seed weight, seed oil percentage, number of leaves, and grain yield (Sepehr & Malakouti, 2004). In the comparison chart (Figures 1 and 2), the highest diameter was observed for treatments in which animal manure was used. The use of manure with chemical fertilizer (Figure 1) and fungi (Figure 2) results in the absorption of most of the elements, and eventually created a thicker head with an effect on growth.

### 3.9 Heading stage

**FIGURE 3:**
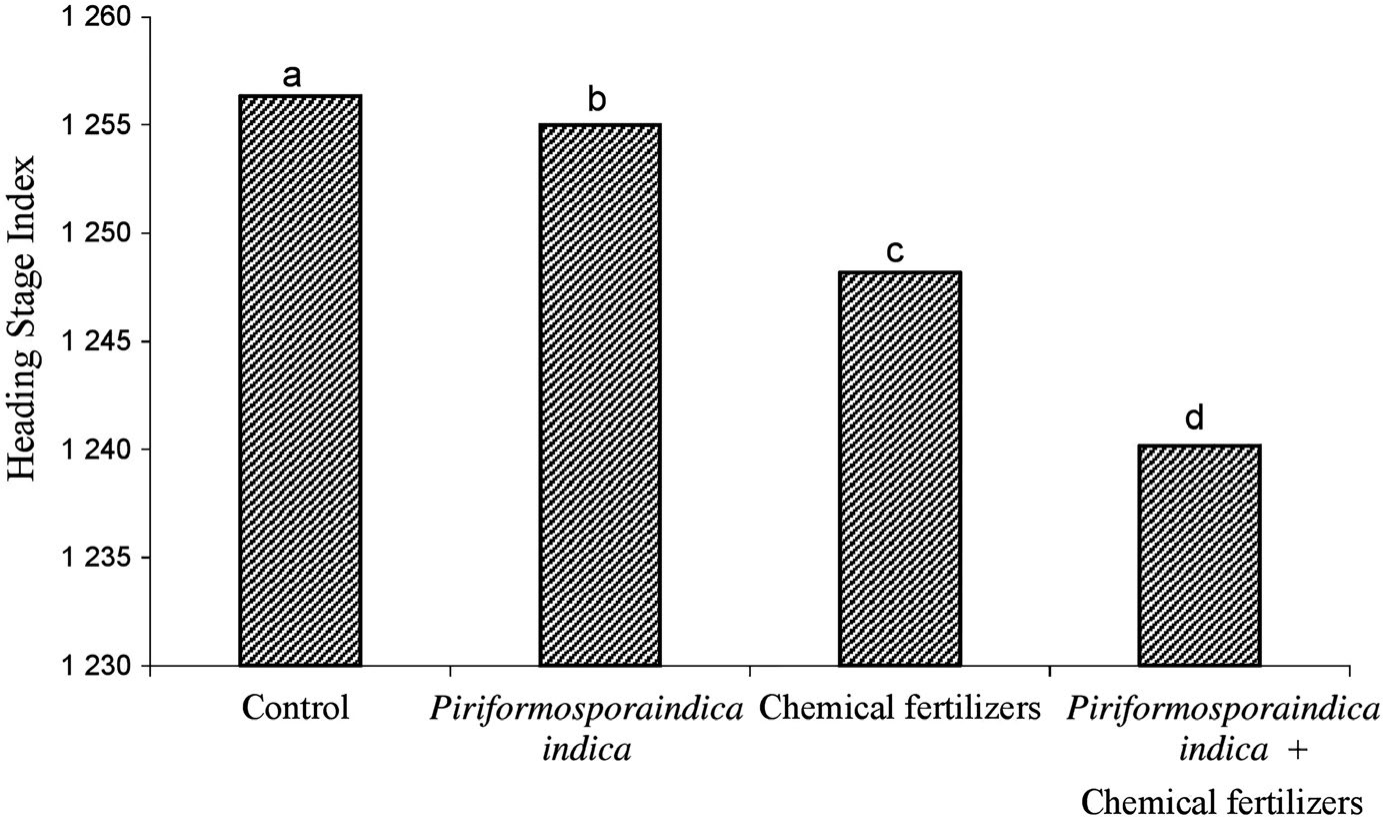
Shows the effect of *P. indica* and chemical fertilizer on head diameter index in sunflower plant

**FIGURE 4:**
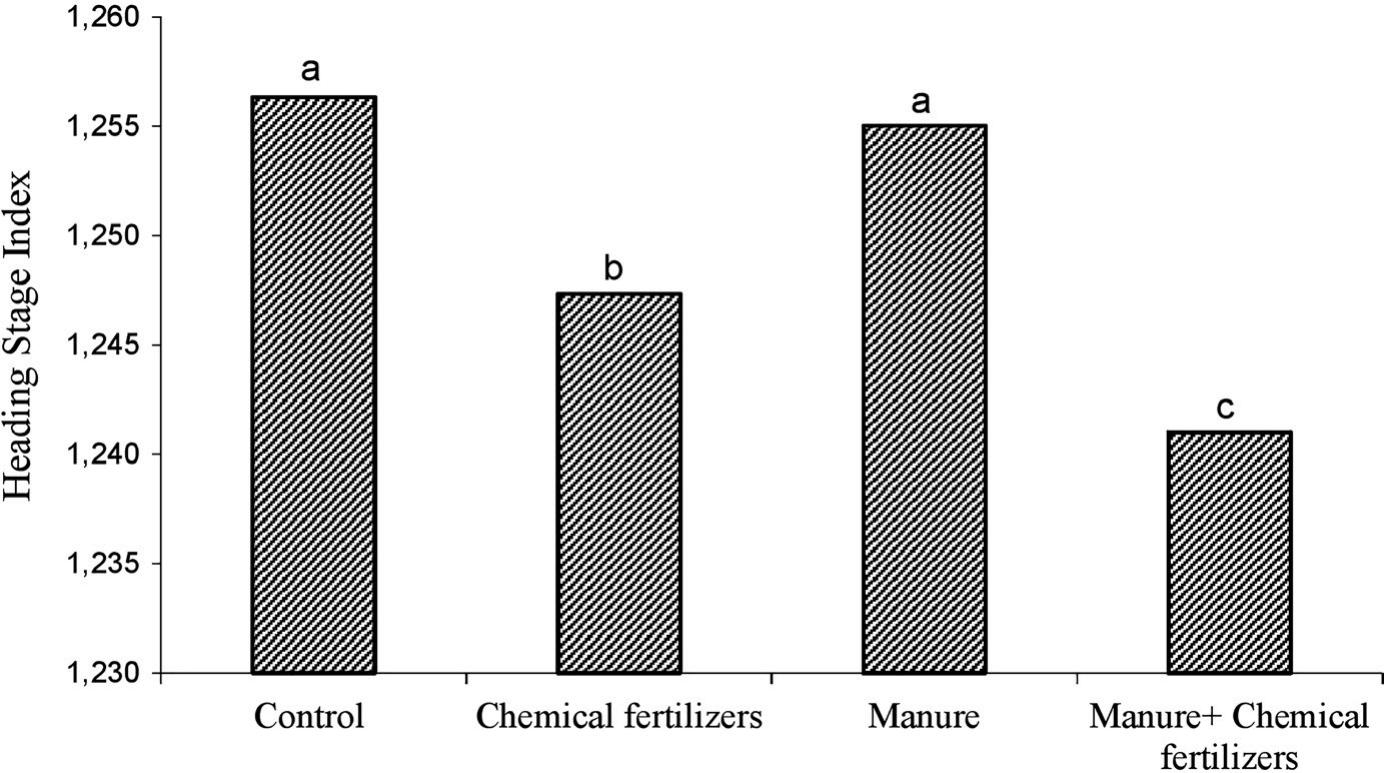
Shows the effect of chemical fertilizer and manure on heading index in sunflowers

The heading index, as the growth index, depends on the amount of nutrition during the plant growth period. The graph for comparison of the average application of chemical fertilizer with fungal inoculum (Figure 3) and the treatment of chemical fertilizer with manure (Figure 4) showed that the minimum time for the appearance of the head belongs to the plants which were fertilized with chemical fertilizers, fungi (Figure 3) and chemical fertilizer and animal manure (Figure 4). Nutrition plays an effective role in emergence of head (Marius et al., 2005). By colonization of plants, P. indica fungi create a way to absorb more elements and then increase the growth rate (Padash et al., 2016). The more the plant absorbs the growth elements in the early stages of growth, the sooner it will end the vegetative phase and enter the reproductive phase (Hafeez, Safdar, Chaudhry, & Malik, 2004).

## 4 CONCLUSION

Soil fertilization management using organic fertilizers such as biologic fertilizers and animal manure can be very important for maximizing the efficiency and sustainability of the soil. On the other hand, chemical treatments cannot fully satisfy the need for sunflower fertilizer in the short term because of the gradual release of nutrients. The fungus P. indica can be a suitable replacement for most biological fertilizers in the future because of its high ability to coexist with plants. The combined method is better than the rest of the treatments. Therefore, for most indexes, the use of all three factors had the best result. Therefore, the combined provision of nutrients using biologic fertilizers and manures, while compensating for the deficiency of nutrients, maintains soil fertility, and sustained production. It seems that the inoculation of grain with P. indica, along with the optimum use of manure, increases nutrient uptake, improves the growth rates and developmental stages of the plant, and ultimately increases the grain yield of sunflower. Despite of the low colonization in plants which were treated with fungi with chemical fertilizer, the highest yield was seen in this treatment. It showed that the P. indica fungi also have its effect on its low percentages. We hope to have a way to make these fungi be widely used in sustainable agriculture in the future.

## CONFLICT OF INTEREST

The authors have declared no conflict of interest.

## AUTHORS CONTRIBUTION

**Siamak Eliaspour** involved in investigation, conceptualization, methodology, software, data analysis, and writing. **Raouf Seyed Sharifi** involved in software, data analysis, validation, and writing. **Ali Shirkhani** involved in writing—review and editing.

## ETHICAL APPROVAL

This study does not involve any human or animal testing.

## INFORMED CONSENT

Written informed consent was obtained from all study participants.
